# Antemortem network analysis of spreading pathology in autopsy-confirmed frontotemporal degeneration

**DOI:** 10.1093/braincomms/fcad147

**Published:** 2023-05-12

**Authors:** Min Chen, Sarah Burke, Christopher A Olm, David J Irwin, Lauren Massimo, Edward B Lee, John Q Trojanowski, James C Gee, Murray Grossman

**Affiliations:** Department of Radiology, University of Pennsylvania, Philadelphia, PA 19104, USA; Department of Neurology, Penn Frontotemporal Degeneration Center, University of Pennsylvania, Philadelphia, PA 19104, USA; Department of Radiology, University of Pennsylvania, Philadelphia, PA 19104, USA; Department of Neurology, Penn Frontotemporal Degeneration Center, University of Pennsylvania, Philadelphia, PA 19104, USA; Department of Bioengineering, Bioengineering Graduate Group, University of Pennsylvania, Philadelphia, PA 19104, USA; Department of Neurology, Penn Frontotemporal Degeneration Center, University of Pennsylvania, Philadelphia, PA 19104, USA; Department of Neurology, Neuroscience Graduate Group, University of Pennsylvania, Philadelphia, PA 19104, USA; Department of Pathology and Laboratory Medicine, Center for Neurodegenerative Disease Research, University of Pennsylvania, Philadelphia, PA 19104, USA; Department of Neurology, Penn Frontotemporal Degeneration Center, University of Pennsylvania, Philadelphia, PA 19104, USA; Department of Pathology and Laboratory Medicine, Center for Neurodegenerative Disease Research, University of Pennsylvania, Philadelphia, PA 19104, USA; Department of Pathology and Laboratory Medicine, Center for Neurodegenerative Disease Research, University of Pennsylvania, Philadelphia, PA 19104, USA; Department of Radiology, University of Pennsylvania, Philadelphia, PA 19104, USA; Department of Bioengineering, Bioengineering Graduate Group, University of Pennsylvania, Philadelphia, PA 19104, USA; Department of Neurology, Penn Frontotemporal Degeneration Center, University of Pennsylvania, Philadelphia, PA 19104, USA; Department of Bioengineering, Bioengineering Graduate Group, University of Pennsylvania, Philadelphia, PA 19104, USA; Department of Neurology, Neuroscience Graduate Group, University of Pennsylvania, Philadelphia, PA 19104, USA

**Keywords:** network analysis, multimodal imaging, autopsy-confirmed, FTD, FTLD

## Abstract

Despite well-articulated hypotheses of spreading pathology in animal models of neurodegenerative disease, the basis for spreading neurodegenerative pathology in humans has been difficult to ascertain. In this study, we used graph theoretic analyses of structural networks in antemortem, multimodal MRI from autopsy-confirmed cases to examine spreading pathology in sporadic frontotemporal lobar degeneration. We defined phases of progressive cortical atrophy on T_1_-weighted MRI using a published algorithm in autopsied frontotemporal lobar degeneration with tau inclusions or with transactional DNA binding protein of ∼43 kDa inclusions. We studied global and local indices of structural networks in each of these phases, focusing on the integrity of grey matter hubs and white matter edges projecting between hubs. We found that global network measures are compromised to an equal degree in patients with frontotemporal lobar degeneration with tau inclusions and frontotemporal lobar degeneration-transactional DNA binding protein of ∼43 kDa inclusions compared to healthy controls. While measures of local network integrity were compromised in both frontotemporal lobar degeneration with tau inclusions and frontotemporal lobar degeneration-transactional DNA binding protein of ∼43 kDa inclusions, we discovered several important characteristics that distinguished between these groups. Hubs identified in controls were degraded in both patient groups, but degraded hubs were associated with the earliest phase of cortical atrophy (i.e. epicentres) only in frontotemporal lobar degeneration with tau inclusions. Degraded edges were significantly more plentiful in frontotemporal lobar degeneration with tau inclusions than in frontotemporal lobar degeneration-transactional DNA binding protein of ∼43 kDa inclusions, suggesting that the spread of tau pathology involves more significant white matter degeneration. Weakened edges were associated with degraded hubs in frontotemporal lobar degeneration with tau inclusions more than in frontotemporal lobar degeneration-transactional DNA binding protein of ∼43 kDa inclusions, particularly in the earlier phases of the disease, and phase-to-phase transitions in frontotemporal lobar degeneration with tau inclusions were characterized by weakened edges in earlier phases projecting to diseased hubs in subsequent phases of the disease. When we examined the spread of pathology from a region diseased in an earlier phase to physically adjacent regions in subsequent phases, we found greater evidence of disease spreading to adjacent regions in frontotemporal lobar degeneration-transactional DNA binding protein of ∼43 kDa inclusions than in frontotemporal lobar degeneration with tau inclusions. We associated evidence of degraded grey matter hubs and weakened white matter edges with quantitative measures of digitized pathology from direct observations of patients’ brain samples. We conclude from these observations that the spread of pathology from diseased regions to distant regions via weakened long-range edges may contribute to spreading disease in frontotemporal dementia-tau, while spread of pathology to physically adjacent regions via local neuronal connectivity may play a more prominent role in spreading disease in frontotemporal lobar degeneration-transactional DNA binding protein of ∼43 kDa inclusions.

## Teaser

Chen *et al*. utilize graph-theoretic analyses of antemortem, multimodal MRIs to examine spreading pathology in structural networks of those with autopsy-confirmed frontotemporal lobar degeneration with either tau or transactional DNA binding protein of ∼43 kDa inclusions. They report distinct patterns of long-range and short-range network degradation between the two pathologies, suggesting different mechanisms for their spread.

## Graphical Abstract

**Graphical abstract:**
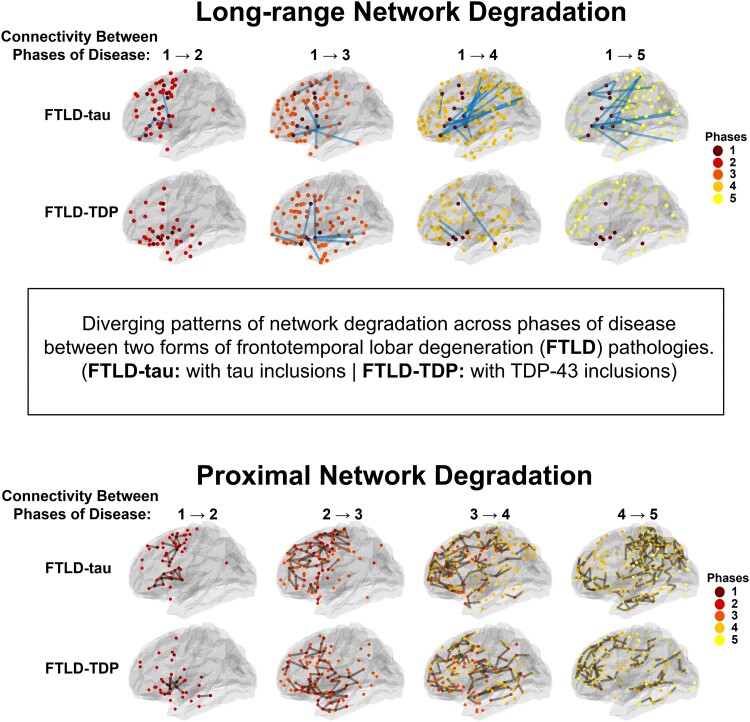

## Introduction

Misfolded proteins found in neurodegenerative diseases have been studied extensively in animal models, and recent emphasis has been placed on understanding the basis for the spreading of this pathology.^1–5^ Animal studies find evidence of propagation of pathogenic protein inclusions, but the anatomical projections vulnerable to specific proteinopathies are not entirely clear. While this foundational work in animals is critical, the brain of a human differs in fundamental ways from that of a mouse.^6^ It is thus essential to examine whether similar mechanisms of disease spread are present by assessment of humans. This work depends in part on whole-brain studies of pathology in humans, and while highly informative,^7,8^ these studies are extremely rare since they are so time-consuming and difficult to execute. Here, we used network science to examine patterns of disease spread by interrogating multimodal, antemortem MRIs of autopsy-confirmed humans with frontotemporal lobar degeneration (FTLD) pathology and used pathologic observations to validate our MRI findings.

Although uncommon, sporadic FTLD is an important cause of early-onset dementia.^9,10^ Two forms of FTLD pathology account for the vast majority of cases with clinical frontotemporal dementia (FTD). These include the accumulation of misfolded tau (FTLD-tau) or misplaced nuclear transactional DNA binding protein of ∼43 kDa (FTLD-TDP).^11^ Phases of progressive cortical atrophy in FTLD-tau and FTLD-TDP have been examined in postmortem series. Adopting the staging approach developed by Braak,^7,8^ this work examines the presence or absence of pathology and its severity, and uses this to construct a series of stages of the hypothesized spread of disease. Reports describe the densest burden of pathology—regions where pathology has accumulated for the longest period of time—in frontal and temporal neocortical and limbic regions in sporadic FTLD.^12–14^ It has been postulated that specific brain regions in sporadic neurodegenerative disease are selectively vulnerable to protein misfolding, and these earliest manifestations of accumulating pathology may serve as epicentres of disease.^15–18^ Epicentres may guide the subsequent anatomic distribution of disease spread.^19^ We and others find some differences between FTLD-tau and FTLD-TDP^20^ that may influence the spread of disease, including relatively deeper cortical layer pathology^21^ and additional glial pathology in white matter (WM)^20,22,23^ in FTLD-tau compared to FTLD-TDP.

A major impediment to studies of spreading disease in autopsied humans is that tissue sampling is typically sparse, thereby limiting the ability to observe all brain regions that may be implicated in spreading pathology. One way to circumvent this limitation is through strategic probing of hypothesized regions implicated in spreading disease. Investigators thus examined spread of tau from the cerebrum into hypothesized brainstem regions in behavioural variant frontotemporal dementia (bvFTD) and corticobasal syndrome.^24^ Reasoning that these clinical syndromes would implicate different descending pathways, they found relatively distinct distributions of tau pathology in the corticospinal tract for corticobasal syndrome and in the prefrontopontine pathway in bvFTD.

In the present study, we adopt the approach of studying the entire cerebrum in antemortem MRI imaging of autopsy-confirmed FTLD-tau and FTLD-TDP. While MRI does not provide pathologic information, rare studies appear to show reasonable correspondence between each type of FTLD pathology and MRI atrophy,^25,26^ and a very small number of studies has examined direct comparisons of regional MRI atrophy with regional pathology burden.^27–29^ The greatest amount of reduced cortical volume seen on antemortem MRI in sporadic FTLD is observed in frontal and temporal grey matter (GM) regions.^25,30,31^ Subsequent phases of disease may be associated with other, more posterior GM regions, and these show more modest reductions of cortical volume.^31^

Network science offers a novel approach to studying the spread of pathology throughout the entire cerebrum in neurodegenerative disease. Network models are designed to characterize complex structures composed of ‘nodes’ (GM regions) connected by ‘edges’ (WM projections between nodes).^32–34^ Moreover, a special class of nodes known as ‘hubs’ may play a more prominent role in neurodegenerative disease. Hubs may have biological importance in that they represent highly connected regions associated with many edges, which may be impacted early in disease due to high levels of WM connectivity and associated heavy burden of informational traffic.^33^ Previous work has suggested disruption of network connectivity, typically using resting state or functional connectivity in clinically defined patients. Instead, we examine spreading pathology through structural networks composed of GM nodes integrated by WM projections between nodes, and how brain connectivity changes as a function of the phase of disease. We decided to use structural networks since we could verify pathology in implicated GM nodes and WM edges. We are unaware of previous work examining network integrity in patients with autopsy-confirmed FTLD-tau and FTLD-TDP, and this is crucial because each pathology may impact network integrity and disease spread in a distinct way, regardless of the clinical phenotype.

We hypothesized that local network features would differ between FTLD-tau and FTLD-TDP, suggesting structural connectivity patterns consistent with each pathology. Given the strategic location of tau pathology preferentially in deeper cortical layers with long-distance projections,^21^ we expected degraded hubs in FTLD-tau epicentre regions to have a greater impact on network integrity earlier in the course of the disease. Moreover, given that deep-layer pathology is also the source of lengthier WM projections, and evidence for greater glial pathology in WM in FTLD-tau,^20–23^ we hypothesized that the connectivity network in FTLD-tau, compared to that of FTLD-TDP, would reveal phase-by-phase transitions driven in part by degeneration of the long-distance WM projections, resulting in overall weaker connections between epicentre nodes and distal regions affected in subsequent phases. Given the preferential location of FTLD-TDP pathology in more superficial GM layers with shorter, proximal projections, we hypothesized that phase-to-phase progression of the disease would be associated with spread to GM regions adjacent to areas of earlier disease.

## Materials and methods

### Subjects

We retrospectively analysed data from 41 autopsy-confirmed patients (28 FTLD-tau, 13 FTLD-TDP) and 27 healthy controls (HCs) available in the Penn Integrated Neurodegenerative Disease Database^35^ at the Penn Frontotemporal Degeneration Center. Pathological diagnosis was made by expert pathologists (E.B.L. and J.Q.T.) using published criteria^11,36,37^ and established procedures.^10^ Inclusion in the study required antemortem MRI with both a T_1_-weighted scan and a diffusion-weighted imaging (DWI) scan with 30 or more directions. Inclusion criteria also included images passing visual inspection for adequate raw image and image segmentation quality. An experienced cognitive neurologist (M.G., D.J.I. and L.M.) evaluated each patient within 1 year of the scan date and diagnoses were reviewed at weekly multidisciplinary consensus meetings. Exclusion criteria included co-pathology greater than minimum burden, evidence of stroke, head injury, infection, immune-mediated disorder, intracranial mass or hydrocephalus. Patients with FTLD-associated genetic mutations may have somewhat different clinical, pathological and imaging characteristics relative to those with sporadic FTLD,^30,38,39^ so we excluded participants with known mutations to avoid these potential confounding factors. We also excluded patients with amyotrophic lateral sclerosis without a cognitive deficit according to the Edinburgh Cognitive and Behavioral ALS Screen^40^ or reported cognitive and/or social difficulty. Controls matched patients for age, education and sex, and were negative for neurological or psychiatric history. Many subjects in the FTLD-tau and FTLD-TDP cohorts predated the standard use of the Clinical Dementia Rating, thus the Mini-Mental State Examination was used as a measure of clinical disease severity. Table 1 summarizes the characteristics of the patient groups. Chi-squared tests of sex ratios and initial phenotype showed no difference between groups (*P* > 0.05). *T*-tests found no difference between groups in years of education, age at MRI, or disease duration, or disease severity (*P* > 0.05). All procedures for patients and HCs were performed following an informed consent procedure in accordance with the Declaration of Helsinki and approved by the Institutional Review Board at the University of Pennsylvania.

**Table 1:** Demographics, clinical phenotypes and pathology subtypes for FTLD-tau and FTLD-TDP cohorts

	FTLD-tau	FTLD-TDP	*P*-value
*N*	28	13	
Age at MRI (years)	67.5 [63.8–71.3]	63.2 [59.0–65.0]	0.09
Sex = male (%)	18 (64.3)	7 (53.8)	0.77
Education (years)	16.7 [16.0–18.0]	15.6 [14.0–18.0]	0.18
Disease duration	4.79 [2.75–6.25]	3.00 [1.00–4.00]	0.08
Disease severity (MMSE)	24.75 [21.5–28.5]	22.7 [20–28]	0.32
Presenting phenotype			0.20
Progressive aphasia	5	1	
Behavioural variant	9	8	
Motor disorder	14	4	
Pathology subtypes			
TDP-A		4	
TDP-B		3	
TDP-C		3	
TDP-E		3	
PSP	13		
CBD	6		
PiD	9		

Descriptive statistics for groups at MRI visit. Mean and interquartile range [mean (IQR)] are provided. Presenting clinical phenotypes were obtained from the clinical records. If several phenotypes were identified throughout life, then the earliest or the most consistently identified with a progressing disease course is listed here. Pathology classifications were confirmed at autopsy and further characterized into subtypes: progressive supranuclear palsy (PSP), corticobasal degeneration (CBD), Pick’s disease (PiD) for FTLD-tau and TDP types A, B, C and E for FTLD-TDP. MMSE, Mini-Mental State Examination.

### Data acquisition and preprocessing

High-resolution volumetric T_1_-weighted MRI and DWI were collected from a SIEMENS 3.0T Tim Trio scanner. T_1_-weighted volumes were acquired using magnetization-prepared rapid acquisition with gradient echo sequences. For most participants (HC *N* = 27, FTLD-tau *N* = 27 and FTLD-TDP *N* = 11), we used an axially acquired protocol with acquisition parameters: repetition time = 1620 ms; echo time = 3.87 ms; flip angle = 15°; matrix = 192 × 256, 160 slices and resolution = 0.9766 mm × 0.9766 mm × 1.0 mm. Remaining participants (FTLD-tau N = 1 and FTLD-TDP N = 2) were imaged using a sagittal sequence with repetition time = 2300 ms, echo time = 2.91 ms, flip angle = 9°, matrix = 240 × 256, 176 slices, and resolution = 1.055 mm × 1.055 mm × 1.2 mm. T_1_-weighted images were preprocessed using the antsCorticalThickness.sh pipeline.^41^ Briefly, each image was normalized into a standard local template space generated from the open access series of imaging studies dataset.^42^ T1-to-template warps were created using registration with symmetric and topology-preserving diffeomorphic deformations, minimizing bias while capturing the regional deformations present in a dementia sample.^43^ ANTs Atropos then used template-based priors to accurately segment images into six tissue classes (cortex, WM, CSF, subcortical GM, brainstem and cerebellum).^44^

For most participants (HC *N* = 27, FTLD-tau *N* = 28 and FTLD-TDP *N* = 11), a single-shot 30-directional DWI sequence was collected using spin-echo diffusion-weighted echo planar imaging (FOV = 240 mm; matrix size = 128 × 128; number of slices = 70; voxel size = 1.875 mm × 1.875 mm × 2 mm, repetition time = 8100 ms; echo time = 83 ms; fat saturation). Thirty volumes with diffusion weight (*b* = 1000 s/mm^2^) were collected along 30 non-collinear directions, and either 1 or 5 volumes without diffusion weight (*b* = 0 s/mm^2^). For the remaining participants (FTLD-TDP *N* = 2), a 48-directional DWI sequence was collected using spin-echo diffusion-weighted echo planar imaging with 48 volumes with *b* = 1000 s/mm^2^ and 7 unweighted volumes (FOV = 232 mm; matrix size = 116 × 116; number of slices = 69; voxel size = 2 mm× 2 mm× 2 mm, repetition time = 7500 ms; echo time = 70 ms; fat saturation). We used ANTs and Camino^45^ to preprocess the DWI. Motion was corrected with affine registration of each diffusion-weighted image to the mean unweighted image. DTs were computed using a weighted linear least-squares algorithm^46^ in Camino. Distortion was corrected by using ANTs to register the motion-corrected mean B0 image to the T1 image.

### Brain network construction

Brain networks were constructed for each individual by performing deterministic tractography. Briefly, cortical regions of interest (ROIs) were created by running the open source ‘easy_lausanne’ package (https://github.com/mattcieslak/easy_lausanne)^47,48^ on the template used for T1 processing, then spatially normalizing the ‘Lausanne_Scale250’ images from template space to each individual’s T1-native space, using the transforms generated during antsCorticalThickness.sh processing. This scale was selected as the regions are similar in size and large enough to be anatomically meaningful. ROIs were constrained to each individual’s cortex by masking with the cortical segmentation generated with the pseudo-geodesic implementation of ANTs joint label fusion,^49^ based on the MindBoggle brainCOLOR dataset^50^ and regional GM volumes were calculated in each of these ROIs. To minimize biases introduced by spatial normalization, streamlines were generated in native DT space by following the optimal tensor orientation at fixed step intervals (0.5 mm), then each streamline image was normalized to the T1 space using the B0-to-T1 warp. Streamline seed points were generated using the WM segmentation from the labelling procedure, plus connected non-cortical regions with fractional anisotropy (FA) > 0.25; to maximize the likelihood we analysed a reasonable set of streamlines, low FA voxels (FA < 0.20) in WM were removed from seeding. To reduce potential GM atrophy effects on WM measurements, cortical ROIs were dilated by 1 voxel into subcortical GM and CSF to close any holes in ROIs artifactually arising due to extensive atrophy in some patients. Streamlines that crossed into CSF were removed as they were considered biologically infeasible. Networks were calculated in T1-native space to minimize the effects of changes in the cortex on the network measurements. FA was calculated along the length of each streamline, and the median FA of each streamline was calculated. To minimize potential confounds of GM structure on the median FA values, streamline endpoints were defined as the GM/WM boundary. The analysed network measurement for each edge was the mean of the median FAs of all streamlines connecting each pair of nodes. FA measurements were chosen to construct the networks due to their easier interpretability as a quantitative assessment of WM structure integrity in each subject.

### Phases of FTLD-tau and FTLD-TDP disease

Disease phases were established for GM volumes in FTLD-tau and FTLD-TDP following the approach presented by Burke *et al*.^29^ Briefly, volume was calculated in each ROI for all participants. To remove effects of intracranial volume and age from each ROI, *W*-scores^51^ were calculated based on the HC cohort data from Burke *et al.*^29^ (*N* = 170) and then applied to each participant’s data. Supplementary Figure 1 shows the mean *W*-score for each region and pathology in our cohort. To optimize sensitivity to varying amounts of atrophy and avoid floor and ceiling effects, *W*-scores were binarized using a threshold of *W* < −0.5.^29^

**Figure 1:**
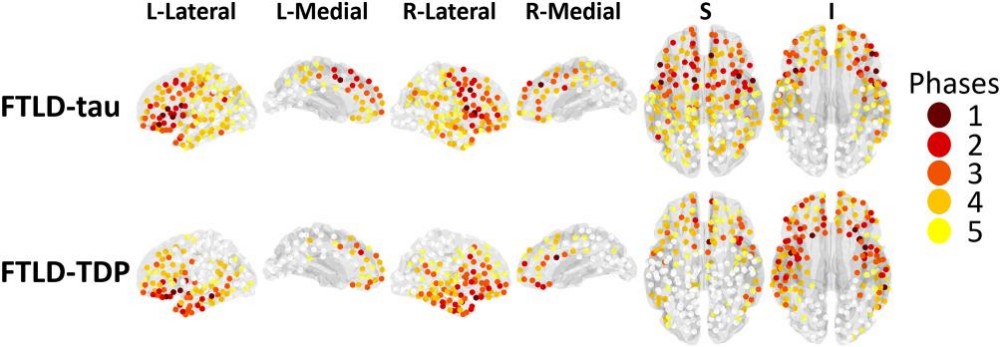
**Distribution of pathology phases in FTLD-tau and FTLD-TDP, as described in Burke *et al*.** ^29^ Phase 1 (epicenter) regions where 90–100% of patients showed reduced cortical volume are coloured dark red. Similarly, Phase 2 (80–89% of patients) are shown in light red; Phase 3 (70–79% of patients) in dark orange; Phase 4 (60–69% of patients) in light orange; and Phase 5 (50–59% of patients) are shown in yellow. The remaining regions (white) exhibited rare atrophy and were not considered in this analysis.

Regions were sorted into phases independently for FTLD-tau and FTLD-TDP. Within each group, phases were defined by the ratio *N*_*a*(*r*)/*N*_*m*, where *N*_*a*(*r*) is the number of patients in the group with atrophy in region *r*, and *N*_*m* = max(*N*_*a*(*r*), *r* = 1, …,448) is the maximum *N*_*a*(*r*) in the group over all regions, where 448 is the total number of cortical regions in the atlas. Phase 1 (epicentre) regions were defined as those with *N*_*a*(*r*) within 90–100% (inclusive) of *N*_*m*. Those within 80–89% were Phase 2; 70–79% Phase 3; 60–69% Phase 4; 50–59% Phase 5. The remaining regions exhibited rare atrophy and were not considered further. Figure 1 shows the ROIs associated with each phase for FTLD-tau and FTLD-TDP.

### Connectivity measures

In brain network science, a *graph* is constructed to represent the connectivity of different regions of the brain. The structural network graph consists of *nodes* representing structural locations in brain, and *edges* representing WM connections between pairs of nodes. Each edge begins and ends at a node, and a *weight* value is assigned to the edge to represent the strength of the connection between the two nodes. From such a graph, we calculate network features which characterize the connectivity patterns and describe the interaction between cortical regions. These descriptors are typically divided into *global* connectivity measures that are calculated from the whole graph, thus producing a single measure for each cerebrum, and *local* connectivity measures, calculated using a subset of nodes that generate a value at each node in the graph. Table 2 provides a short summary for the global and local measures used in this study. The construction of the networks and evaluation of these measures were performed using the Brain Connectivity Toolbox.^55^

**Table 2:** Summary of the global and local connectivity measures used in the network analysis

	Formulation	Interpretation
Global measures		
Global efficiency^52^	Average inverse shortest path in the network.	How parallelizable are the connections in the brain network.
Mean nodal strength^53^	Average sum of weights to each node.	How strongly inter-connected is the brain network.
Local measures		
Nodal strength^53^	Sum of weights to the node.	How well connected is a particular brain region (node) to other brain regions.
Betweenness centrality^54^	Fraction of all shortest paths that pass through the node.	How vital is the brain region (node) for connecting other regions together. (How much traffic goes through this region.)

### Hubs and disease-impacted edges

Using the network graphs, we investigated the relative importance of certain nodes and edges in each disease. *Hub* nodes^56^ can be defined using local connectivity measures to identify nodes that interact strongly with other nodes. We defined hubs as nodes with either a betweenness value or a nodal strength value that is 1 SD above the mean betweenness and nodal strength values, respectively, across nodes from the HC cohort. The betweenness and nodal strength thresholds for determining HC hubs were applied to the FTLD-tau and FTLD-TDP networks to determine disease-specific hubs. We refer to HC hubs that are no longer present in a disease group as *lost* hubs, and hubs found for a disease group, but not present in the HC group as *gained* hubs.

*Weakened* edges are edges in the network that have disrupted WM connectivity in a disease group, relative to HC. We defined weak edges for each individual in each disease group by first calculating a *z*-score for each edge for the subject, and then finding where the *z*-score is −3 or lower. The *z*-score for an edge weight, *w*, is defined as,


(1) $$\begin{array}{c} z=\frac{w-{\bar{w}}_{\mathrm{HC}}}{S_{\mathrm{HC}}} \end{array}$$

where ${\bar{w}}_{\mathrm{HC}}$ and $S_{\mathrm{HC}}$ are the mean and SD of the weights for the same edge across the HC cohort. Missing edges where streamlines could not be found were treated as ‘not-a-number’ (NaNs) in the connectivity network and calculations.

### Proximal connections

Our analyses thus far describe distant connectivity as represented by the edges between nodes that are captured by DWI and often in named fasciculi. We also explore modelling proximal spread by looking at emerging disease in nodes that are directly adjacent to diseased nodes. For each transition between disease phases, we examined the regions where there is significant cortical atrophy that is physically adjacent to a region in a subsequent phase. We define these edges as *proximal spreading connections* in each individual.

### Digital histopathological assessment

We cross-validated our antemortem MRI findings by relating the mean edge *z*-scores with postmortem measurement of percentage of area occupied microscopic pathology burden in GM and WM. We restricted these analyses to subjects who had short disease durations (≤5 years) for whom there is likely to be meaningful accumulation of pathologic burden while minimizing neuronal degeneration and ghost neurons with undetectable tau.^57^ Paraffin-embedded tissue samples were used for digital histology measurements, as described.^58^ We sampled nine cortical regions which are analogous to previously described cortical ROIs and WM regions immediately subjacent to them,^27,28,59^ including anterior insula, orbital frontal cortex, inferior frontal cortex, middle frontal cortex, anterior cingulate cortex, superior-middle temporal cortex, angular gyrus, superior parietal cortex and primary occipital cortex. Sections were stained for phosphorylated-tau (AT8; Thermo Scientific, Waltham, MA, USA) in FTLD-tau and phosphorylated-TDP-43 (p409/410; Millipore, Burlington, MA, USA) in FTLD-TDP, as described.^58^ Whole slide images were acquired in the Penn Digital Pathology Lab on a digital slide scanner (Aperio AT2, Leica Biosystem, Wetzlar, Germany) at ×20 magnification. Images were digitally analysed using QuPath software (version v0.2.0) to calculate the percentage of area occupied by pixels with FTLD-tau or FTLD-TDP from representative GM and adjacent WM, as published previously.^20,27^Supplementary Figure 3 shows representative images of staining for each pathology.

### Statistical analyses

Statistical comparisons between groups were performed using standard univariate statistics for parametric (Welch’s *t*-tests) or non-parametric (Mann–Whitney U) for continuous data or categorical data (*χ*^2^) as appropriate. Global network features across groups were first assessed with an omnibus analysis of variance, followed by pairwise analysis. All statistical and regression analyses were performed using MATLAB (version 2021b).

## Results

### Comparisons of global network measures

**Figure 2:**
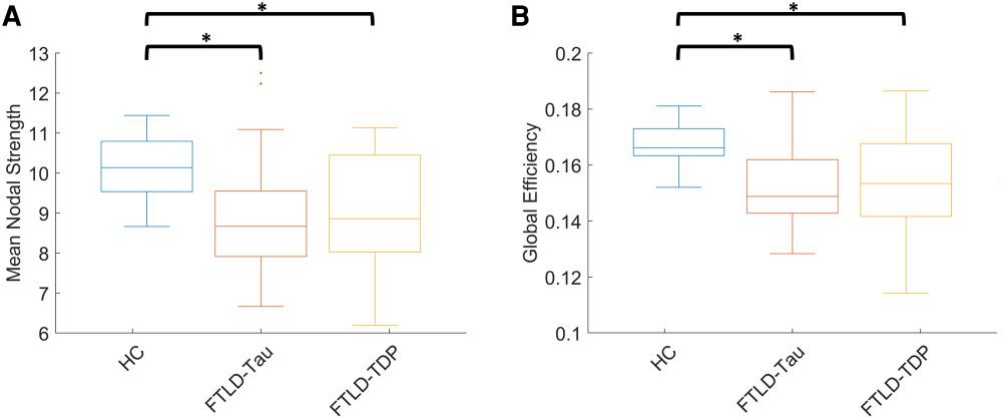
**Comparison of mean nodal strength (MNS) and global efficiency (GE) between disease and control groups.** An analysis of variance showed significant differences among groups in (**A**) MNS [*F*(2,65) = 7.90, *P* < 0.001] and (**B**) GE [*F*(2,65) = 9.31, *P* < 0.001]. Pairwise analysis showed differences between HC and FTLD-tau groups [MNS: *t*(53) = −3.94, *P* < 0.0001; GE: *t*(53) = −4.68, *P* < 0.00001], and between HC and FTLD-TDP groups [MNS: *t*(38) = −3.37, *P* < 0.001; GE: *t*(38) = −3.94, *P* < 0.001]. No significant differences were observed between the two pathology groups for either measure [MNS: *t*(39) = 0.065, *P* > 0.05; GE: *t*(39) = 0.073, *P* > 0.05] (*n* = 27 HC, 28 FTLD-tau, 13 FTLD-TDP).

We first compared the global network measure differences between FTLD-tau, FTLD-TDP and HC cohorts (Fig. 2). Welch’s *t*-test showed significant decreases in both measures when comparing each disease group and the HC group. While FTLD-tau had the lowest nodal strength and global efficiency scores, there was no statistical difference between FTLD-tau and FTLD-TDP.

### Hub analysis

**Figure 3:**
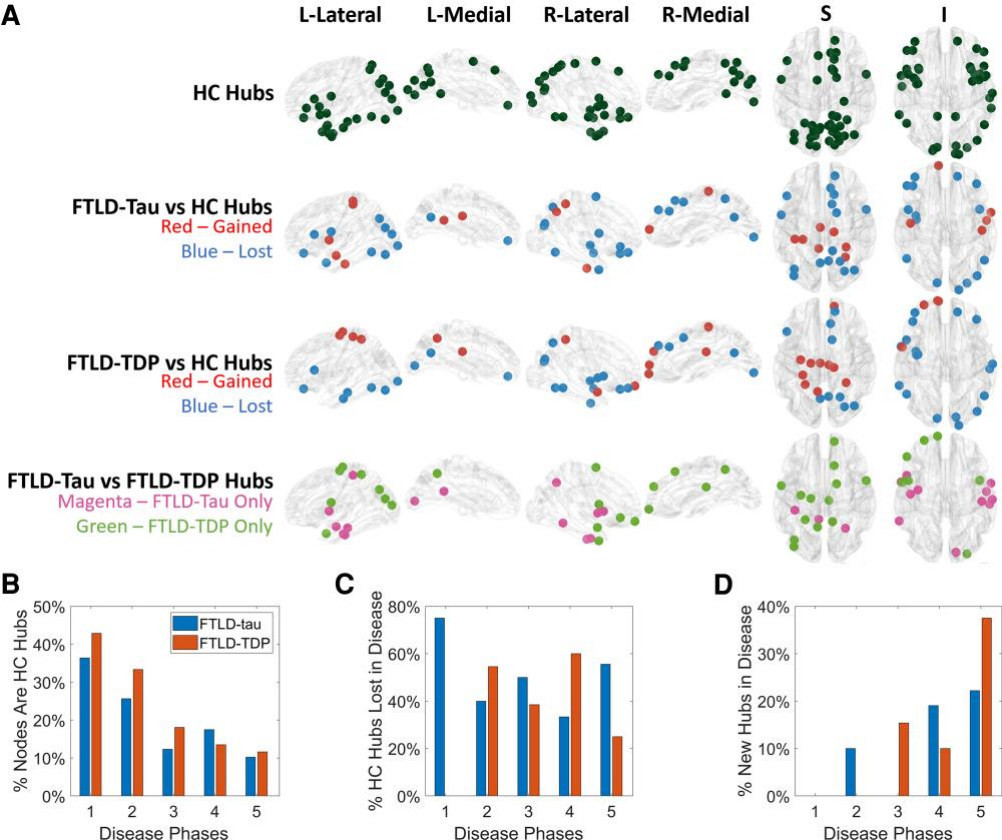
**Visualization of differences in hub distributions between groups and across disease phases.** Shown in (**A**) are the relative locations of control (HC) hubs (dark green), the hubs lost (blue) and gained (red) for each pathology, and hub differences between pathologies (magenta versus green). The graphs below further evaluate these hubs in each phase of FTLD-tau and FTLD-TDP. Shown in **(B)** are per cent of nodes in each phase that are associated with HC hubs that become diseased, (**C**) per cent of HC hubs lost in each phase of each disease and (**D**) per cent of new hubs gained in each disease, relative to total HC hubs in each disease phase. (*n* = 27 HC, 28 FTLD-tau, 13 FTLD-TDP).

Hub analysis was performed on FTLD-tau, FTLD-TDP and HC using the local network measures calculated for each subject. Figure 3A shows the distribution of the hubs found for HC. Hubs detected for our HC cohort follow similarly to previous studies of HC hubs.^60^ Namely, our study detected hubs in the top 5 strongly connected gyri reported previously (superior frontal, superior parietal, precuneus, rostral middle frontal and pre-central gyrus).^60^ Some hubs are also found in frontal and temporal regions thought to be relatively susceptible to early pathology in FTLD-tau and FTLD-TDP.

Figure 3A also illustrates the relative loss of these hubs for FTLD-tau and FTLD-TDP compared to HC. We observed partially distinct sets of hubs lost in FTLD-tau and FTLD-TDP. Many of these are in frontal and temporal regions are associated with atrophy in FTLD spectrum disorders (Fig. 1). Figure 3B shows the relative percent of diseased nodes in each phase in FTLD-tau and FTLD-TDP that are associated with HC hubs. Diseased nodes in FTLD-tau and FTLD-TDP were equally likely to be associated with HC hubs across all phases. Figure 3C shows the percent of these HC hubs that are lost in each phase of disease. We observed that over 70% of the HC hubs were lost in epicentre (Phase 1) regions of FTLD-tau, while none were lost for FTLD-TDP. This emphasizes the early impact of FTLD-tau pathology on strategically located hubs.

Figure 3A also illustrates that some regions appeared to emerge as new hubs during disease progression. One possibility is that these may reflect brain reserve in response to degeneration. Figure 3D shows the relative percent of hubs gained at each phase as a percent of the total number of HC hubs present in the phase. We observed a general trend of new hubs appearing in regions associated with the later phases of disease in both FTLD-tau and FTLD-TDP. From further investigation of which network measure gave rise to these gained hubs (Supplementary Fig. 2), we see that most of the hubs were gained through an increase in betweenness centrality. This is reasonable, since one would not expect overall network connection strength to increase with disease, but the centrality of preserved nodes can increase as other networks paths become compromised.

### Connectivity between phases

**Figure 4:**
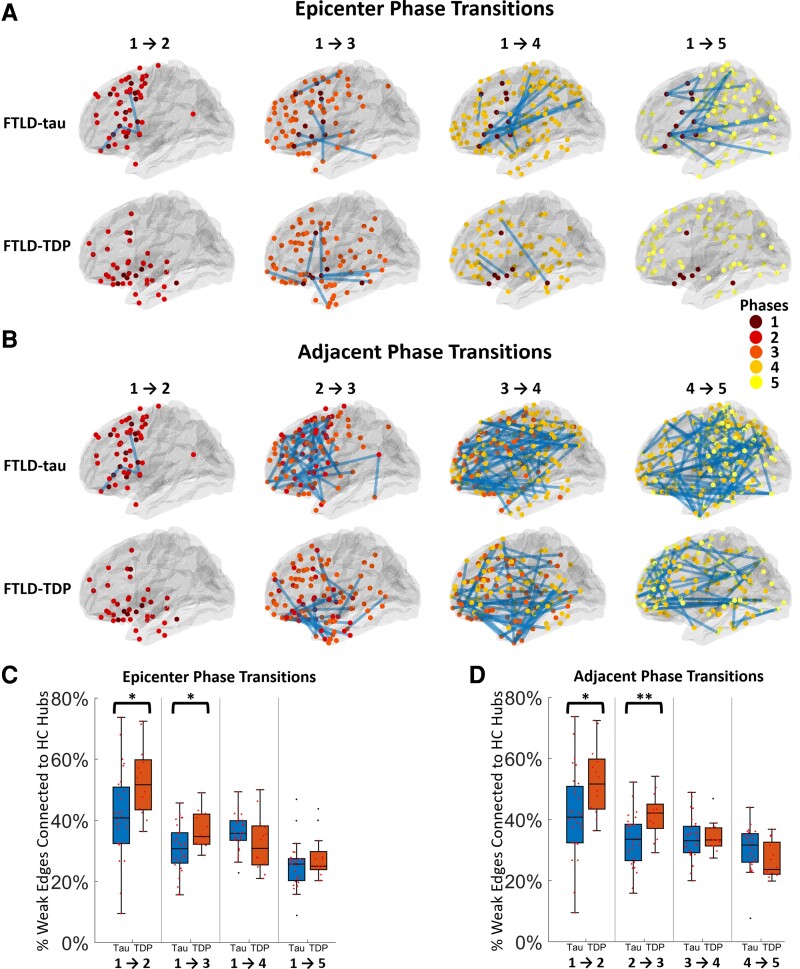
**Network and hub analysis between disease phases.** Weak edges (lines shown) found transitioning between nodes from epicentre to other phases in (**A**) and sequentially from an earlier phase to a subsequent phase in (**B**) for FTLD-tau and FTLD-TDP. Node colours represent regions associated with each phase. Connections are shown as projections onto the left lateral view. Shown in (**C**) and (**D**) are comparisons of the percentage of these weak edges that were connected to HC hubs, per subject, during the transitioning from the epicentre or in adjacent phases, respectively. Asterisks (*P < 0.05 and **P < 0.01) indicate phases with significant differences found between the two pathology groups using a Welch’s *t*-test. (Phases transitions [1–2]: *t*(39) = −2.46, *P* < 0.05; [1–3]: *t*(39) = −2.64, *P* < 0.05; [1–4]: *t*(39) = 1.38, *P* > 0.05; [1–5]: *t*(39) = −1.12, *P* > 0.05; [2–3]: *t*(39) = −3.03, *P* < 0.01; [3–4]: *t*(39) = −0.27, *P* > 0.05; [4–5]: *t*(39) = 1.61, *P* > 0.05; *n* = 28 FTLD-tau, 13 FTLD-TDP).

The mean *Z*-scores between all edges connecting nodes in FTLD-tau and FTLD-TDP were compared across the whole cerebrum using a Welch’s *t*-test, and we found that edges were significantly degraded in FTLD-tau compared to FTLD-TDP (*P* = 0.00096). We then examined how weakened edges are associated with transitions across different phases for each disease by examining edges from the full network connected only to nodes that defined pairs of phases in each disease. Figure 4 shows the weakened edges (blue lines) between epicentre (Phase 1) nodes and nodes in each of the other five phases (Fig. 4A) and between nodes associated with each subsequent phase (Fig. 4B). We observed a clear pattern where FTLD-tau is associated with many additional weakened edges between phases compared to FTLD-TDP. Figure 4C and D shows a subject-level evaluation of weakened edges between the cohorts. We evaluated the number of weakened edges found for each individual (represented by red points) and then used Welch’s *t*-tests to compare the number of weak edges in each subject in FTLD-tau and FTLD-TDP groups between each phase of progressive disease. The Phase 1 epicentre to Phase 2 transitions were found to be significantly reduced in FTLD-tau compared to FTLD-TDP (*α* = 0.05), after normalizing for the number of nodes in each phase. This emphasizes the disproportionate impact of early disease on the integrity of projections in FTLD-tau compared to FTLD-TDP. We also found significantly reduced edges between epicentre to Phase 5 nodes (*α* = 0.05) in FTLD-tau compared to FTLD-TDP. When we examined hubs in the same way, the findings were virtually identical.

### Hub combined with weakened edges

Combining the hub analysis and the weakened edges analysis, we investigated how degraded hub nodes are associated with weakened edges affected by each pathology across phases of progressive disease. Specifically, we assessed the percent of weakened edges that are connected to disease-specific hubs (Fig. 3) for each pair of adjacent phases and from the epicentre to each phase of disease in FTLD-Tau compared to FTLD-TDP. Figure 4C and D shows these comparisons for edges connected to the hubs within each phase. We found that edges associated with diseased hubs are significantly reduced in FTLD-tau compared to FTLD-TDP. This was most evident in the early phases of disease.

### Modelling proximal spread

**Figure 5:**
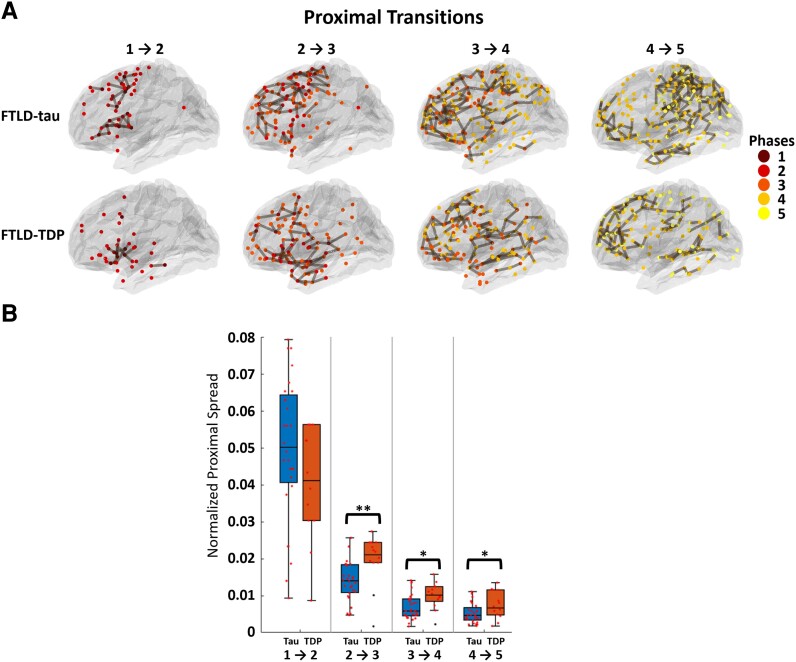
**Analysis of proximal spreading connections by disease phases.** (**A**) A group representation of locally spreading connections between nodes in regions adjacent to diseased nodes for FTLD-tau and FTLD-TDP is shown. Lines show spread from diseased regions in a phase to adjacent regions in a subsequent phase. (**B**) Comparisons of the number of proximal spreading connections among subjects of each group per phase transition, normalized by the total possible connections between phases (product of the number of total regions in each phase) are shown. Asterisks (*P < 0.05 and **P < 0.01) indicate phases with significant differences found between the two pathology groups using a Welch’s *t*-test. (Phases transitions [1–2]: *t*(39) = 1.38, *P* > 0.05 ; [1–3]: *t*(39) = −2.81, *P* < 0.01; [1–4]: *t*(39) = −2.70, *P* < 0.05; [1–5]: *t*(39) = −2.28, *P* < 0.05; *n* = 28 FTLD-tau, 13 FTLD-TDP).

Figure 5A shows the group-level representation of proximal spreading connections for both FTLD-tau and FTLD-TDP. Figure 5B shows a subject-level comparison of the number of these adjacent connections between the two cohorts. For each subject, we calculated the number of proximal spreading connections in each phase transition and then normalized this value by the total possible connections between the two phases (product of the number of regions in each phase). Welch’s *t*-test showed significant differences between patient cohorts for Phase 2 to 3, Phase 3 to 4 and Phase 4 to 5. In general, FTLD-tau and FTLD-TDP had similar levels of proximal spreading degradation in the earliest phase, but in later phases, FTLD-TDP exhibited higher counts of proximal spread than FTLD-tau. This appears to emphasize the relative importance of proximal spread in FTLD-TDP.

### Relating whole-brain antemortem imaging to pathology at autopsy

**Figure 6:**
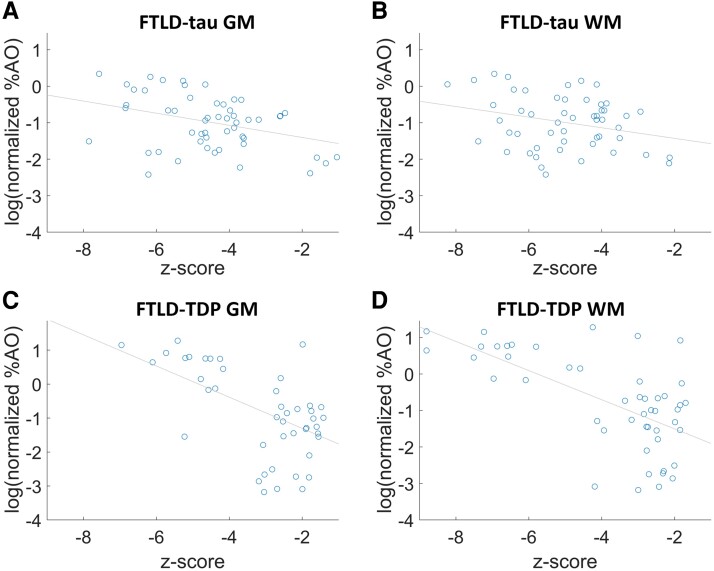
**Relating network analysis with histopathology measurements.** Plots showing the relationship between percentage of area occupied (%AO) for each sampled region in each subject and the average *z*-score of the edges connected to those regions. The analysis was repeated for pathology measurements of (**A**) FTLD-tau GM, (**B**) FTLD-tau WM, (**C**) FTLD-TDP GM and (**D**) FTLD-TDP WM from subjects with five or less years of disease duration. Significant relationships were found for all four analyses when evaluating the linear regression coefficient (**B**) and Pearson’s correlation coefficient (*r*): FTLD-tau GM (*b* = −0.166, *P* = 0.006882; *r* = −0.364, *P* = 0.0069), FTLD-tau WM (*b* = −0.145, *P* = 0.0473; *r* = −0.276, *P* = 0.0474), FTLD-TDP GM (*b* = −0.459, *P* = 9.493e−05; *r* = −0.543, *P* = 9.493e−05), FTLD-TDP WM (*b* = −0.399, *P* = 1.314e−06; *r* = −0.634, *P* = 1.314e−06). (*n* = 28 FTLD-tau, 13 FTLD-TDP).

We evaluated the linear regression coefficient (*b*) between the antemortem MRI findings and postmortem pathology measurements (Fig. 6), and observed significant relationships between percentage of area occupied pathologic burden and the average *z*-score of edges connected to each node as well as the integrity of that GM or WM node. This relationship was found for both FTLD-tau and FTLD-TDP, and for both GM and WM pathology measurements: FTLD-tau GM (*b* = −0.166, *P* = 0.006882), FTLD-tau WM (*b* = −0.145, *P* = 0.0473), FTLD-TDP GM (*b* = −0.459, *P* = 9.493e−05) and FTLD-TDP WM (*b* = −0.399, *P* = 1.314e−06).

## Discussion

Microscopic studies of whole-brain pathology in neurodegenerative diseases are potentially informative in understanding the spread of pathology throughout the cerebrum.^7,8^ Direct studies of whole-brain pathology are very rare and extremely challenging. Here, we combine network science analyses of whole brain, multimodal MRI with analysis of phases of disease progression to study patterns of degraded structural network connectivity in autopsy-confirmed FTLD-tau and FTLD-TDP. While analyses of global indices demonstrated comparably degraded networks in both FTLD-tau and FTLD-TDP, we observed significant differences between groups in analyses using local networks. Specifically, we found greater loss of hubs in frontal and temporal epicentres in the early phases of disease in FTLD-tau than FTLD-TDP. We also found that edges are more degraded in FTLD-tau than FTLD-TDP, particularly early in disease, and that this interferes with the integrity of projections from epicentre hubs to other, distant brain regions in FTLD-tau more than FTLD-TDP. By comparison, spread of disease from diseased regions to adjacent regions in subsequent phases of disease appeared to be more prominent in FTLD-TDP than FTLD-tau. In direct comparisons of MRI and autopsy sampling, we confirmed that network disruption is related in part to the burden of GM and WM pathology in FTLD-tau and FTLD-TDP. We conclude that loss of epicentre hubs and degraded WM projections from these hubs to other, distant brain regions appears to be consistent with distant, axon-mediated disease progression in FTLD-tau, while serial involvement of regions physically adjacent to diseased regions appears to be consistent with proximal disease spread in FTLD-TDP.^1–5^

Our study revealed significant disruption of global network integrity, as measured by *global efficiency* and *mean nodal strength,* in patients with known FTLD-tau or FTLD-TDP pathology. Some studies of clinically diagnosed FTD patients also have observed a variety of compromised global network indices.^61–63^ Reports described reduced global efficiency in non-fluent/agrammatic variant primary progressive aphasia (naPPA) relative to bvFTD^64^ and degradation of several global indices in svPPA,^65^ but another study reported little compromise of global network measures in bvFTD.^66^ While informative, the basis for reported findings must be interpreted cautiously since pathology is unknown in most published work assessing global network indices in clinically diagnosed patients. While we found disrupted global network indices, we did not find that FTLD-tau or FTLD-TDP globally degrade the network integrity of the human brain in a distinctive manner. One difference between our study and most previous work is that we interrogated structural networks rather than functional networks, particularly because this allowed us to directly examine pathology in network components, and future work using functional connectivity in autopsy-confirmed cases would be valuable to validate functional network assessments. It is also possible that each pathology may have a selective impact on global network indices other than the ones we probed, and additional work is needed to assess other global network characteristics.

By comparison, we found that analyses based on local network indices of *betweenness centrality* and *nodal strength* in humans show differences between autopsy-confirmed cases of FTLD-tau and FTLD-TDP. Spreading pathology has been well studied in animal models.^67–70^ Differences in local network indices have been reported in clinically diagnosed patients, often guided by the specific anatomic distribution of disease. Analyses of svPPA and bvFTD showed that identification of individual epicentres can predict patterns of longitudinal decline.^71^ Distinct patterns of breakdown in local network functional connectivity have been described in naPPA compared to svPPA,^72^ including local network disruption in naPPA involving nodal degree, local efficiency and clustering coefficient most prominently in the frontal lobe, while svPPA may have similar local network disruption in the temporal lobe.^61^ Some work focusing on predefined functional networks reported a correlation between nodes implicated in the speech/language network in HC and degraded integrity in the speech/language network of patients with naPPA, and regions with the shortest paths to the epicentre showed the greatest longitudinal decline.^73^ While there is a statistical association between patients with naPPA who are more likely to have FTLD-tau pathology and patients with svPPA who are more likely to have FTLD-TDP pathology,^74^ an autopsy assessment is needed to rule out concomitant co-pathology frequently found in clinically diagnosed cohorts.^9,10^

In our study, differences in local connectivity measures appeared to compromise network structure in somewhat different ways, depending on the specific pathology, and this may impact the spread of pathology in FTLD-tau compared to FTLD-TDP. Consider first compromised hubs. Hubs are thought to play an important role in cerebral networks because many edges connect with this subset of nodes, and this network of highly connected ‘rich-club’ nodes may play a relatively important role in cognitive functioning.^60^ Significant loss of hubs has been observed in functional networks of clinically defined FTD patients, particularly in frontoinsular regions that may serve as epicentres in some patients.^62^ We observed here that roughly equal proportions of hubs identified in HC are lost in FTLD-tau and FTLD-TDP. However, degraded HC hubs in FTLD-tau tended to be lost most prominently among those implicated in early phases of disease, and appeared to be associated anatomically with FTLD-tau epicentre regions. We observed a difference in hub loss locations between the two disease groups, where FTLD-Tau showed specific hub loss in middle frontal and parietal regions, while FTLD-TDP showed specific loss were centred around the lateral temporal lobe. It is possible that hubs in FTLD-tau epicentres are relatively vulnerable to disease because of their cytoarchitectonic location in deeper cortical layers enriched for cortical pyramidal neurons.^21^ This effect may be exacerbated by the high metabolic activity associated with the heavier ‘traffic’ that may be found in these hubs,^33^ although this would not explain degraded hubs that tend to occur in later phases of disease in FTLD-TDP. The degradation of hubs in FTLD-tau may differ from that in FTLD-TDP where pathology tends to impact superficial cortical layers and thus may have different, possibly reduced consequences and a different temporal course.

Consider next changes in edges in FTLD-tau and FTLD-TDP. We observed significantly weakened edges in FTLD-tau compared to FTLD-TDP. We observed these differences in edges implicated in phase-by-phase transitions, and differences in edges involved in the projections between epicentre regions and each of the later phase regions. Weakened edges were most prominent in earlier phases of disease in FTLD-tau, but there were no weakened edges in the transition from Phases 1 to 2 in FTLD-TDP, and subsequent phase transitions revealed fewer weakened edges in FTLD-TDP than FTLD-tau. This appears to be consistent with the observation that tau pathology tends to be more prominent in deeper cortical layers that are the source of long-tract projections that provide connectivity to distant areas.^21^ From this perspective, diseased hubs, particularly in epicentre regions in FTLD-tau, may interrupt networks that support critical long-tract projections due in part to the cytoarchitectonically deeper locus of FTLD-tau pathology, and this may yield a greater impact on network integrity early in the course of disease. Our analyses also showed that edges appear to be disproportionately weakened compared to hubs in FTLD-tau, and this may be due in part to greater independent WM pathology in these cases.^20–23^ These WM effects may differ or be additive to the effects of Wallerian degeneration that may scale with GM pathology burden regardless of the underlying pathology. We interpret weakened edges during phase transitions to be consistent with distant, axon-mediated spread of pathology that is more prominent in FTLD-tau than FTLD-TDP.

We also examined the spread of pathology from diseased regions to physically adjacent regions in subsequent phases of disease to model microscopic, short-range connectivity spread. We found that spread of pathology to adjacent nodes was more prominent in FTLD-TDP than FTLD-tau. FTLD-TDP pathology tends to involve more superficial cortical layers that are typically associated with shorter projections to proximal regions.^21^ This may result in part in the relatively reduced degradation of hubs and networks in FTLD-TDP, particularly early in disease, since superficial pathology may relatively spare the population of neurons in deeper cortical layers that may be critical for a broader impact on network integrity. Also, there is relatively less glial WM pathology in FTLD-TDP compared to the high burden of glial tau inclusions in FTLD-tau,^20–23^ and this may result in less interruption of longer WM projections. Additional work is needed to examine these findings in network-based analyses of connectivity in high-resolution, whole brain, microscopic tissue samples.

We also observed an association between pathology and imaging studies in FTLD-tau and FTLD-TDP. In a subset of patients with available quantitative digital pathology, we found that WM pathology burden was related to weakened edges, and that GM pathology was related to nodal integrity. In another study assessing graph-theoretical analyses to model microscopic disease progression in FTLD using digital histopathology data, we found that WM pathology correlates with a graph metric of network spread and that WM pathology has a mediation effect on distant projections between GM regions in FTLD-tau but not FTLD-TDP.^75^ These observations provide some independent validation of degraded nodes and edges that contribute to interrupted structural networks in FTLD-tau and FTLD-TDP.

Several caveats should be kept in mind when considering our findings. Although FTLD is a rare condition, and multimodal imaging in autopsy-confirmed cases is even rarer, our cohort is relatively small. Additional studies are needed to confirm our findings in larger groups of patients. We stratified our patients on the basis of pathology, and we were very careful to omit cases with co-pathology. Since phases of progressive disease on MRIs of autopsied cases showed relatively similar patterns across different sub-forms of FTLD-tau and FTLD-TDP pathology,^29^ we attempted to improve power by grouping across the different sub-forms of FTLD-tau and FTLD-TDP pathology. Nevertheless, there may be differences in patterns of spreading pathology in different FTLD-tau and FTLD-TDP subgroups,^76^ and additional work is needed to study each pathologic subgroup to assess the generalization of our findings across each class of pathology. Limitations in power likewise prevented us from looking at phenotype subgroups, and additional work is needed to examine PPA and bvFTD phenotypes within each pathologic group. In this work, we pursued a pseudo-longitudinal approach to determine phases of disease progression in each group. However, our cross-sectional data limits our ability to make true longitudinal assessments of the disease progression. Only a limited number of longitudinal imaging assessments were available in this autopsy cohort, and additional work is needed to examine longitudinal MRI changes in an autopsy-proven cohort. The lack of *in vivo* pathology markers in FTLD also poses a challenge. In this work, we infer the presence of pathology through MR measurements of cortical atrophy. This assumption can be complicated by other sources of atrophy such as gliosis or spongiosis of the cortex, which needs to be explored in further studies. Future longitudinal studies of *in vivo* imaging measurements with available clinical or behaviour assessments of disease progression can also help validate the use of cortical atrophy measurements for our analysis. We studied this cohort with a limited number of global and local network indices to demonstrate the principle that pathology may differentially impact network integrity, and additional work is needed to apply a more comprehensive set of global and local network indices in FTLD spectrum disorders. We interrogated structural networks rather than functional networks because it is possible to obtain independent evidence for compromised network integrity in GM and WM structures contributing to a network from autopsy studies. Nevertheless, it would be valuable to confirm our findings using functional networks in autopsy-confirmed cases. One limitation of the diffusion technique used in this study is its insensitivity to u-fibres, thus limiting our ability to detect shorter-range fibres in general. However, we believe that this limitation is not inherently biased towards either FTLD-tau or FTLD-TDP pathology. Thus, the limited shorter projections we can detect in the data should still offer a valid comparisons between the groups. One advantage of studying an autopsy-confirmed cohort is that we can verify our *in vivo* methodology against actual pathology burden. The threshold used for the *W*-score to establish phases of disease, while relatively liberal, was determined in our previous study,^29^ where we verified that pathology burden aligned with the phases of disease. Additional work is needed with larger samples to assess other statistical thresholds.

With these caveats in mind, our network-based analyses of an autopsy-confirmed FTLD cohort suggest that there are substantial differences in local network degeneration that are associated with FTLD-tau compared to FTLD-TDP. These include both the greater loss of hubs in FTLD-tau, particularly in association with epicentres implicated in earlier phases of disease, and phase-by-phase degeneration of WM projections between hubs in epicentre regions and regions that become compromised later in the course of disease. These findings are consistent with the possibility that spreading pathology in FTLD-tau is mediated in part by axon-mediated spread to distant brain regions. By comparison, we observed spreading pathology in a phase-by-phase manner in FTLD-TDP from earlier diseased regions to physically adjacent regions in subsequent phases. This appears to be more consistent with proximal spread of pathology.

## Supplementary Material

fcad147_Supplementary_Data

## Data Availability

With the approval of our data-sharing committee and a Materials Transfer Agreement, we are pleased to make available anonymized data to qualified scientists who have appropriate approvals from their institution to perform studies in humans. Software developed for the analysis in this work can be downloaded from the following repository: https://github.com/dontminchenit/mBIN.

## Abbreviations

bvFTD =: behavioural variant frontotemporal dementia
DWI =: diffusion-weighted imaging
FTD =: frontotemporal dementia
FTLD =: frontotemporal lobar degeneration
GM =: grey matter
naPPA =: non-fluent/agrammatic variant primary progressive aphasia
svPPA =: semantic variant primary progressive aphasia
TDP-43 =: transactional DNA binding protein of ∼43 kDa
WM =: white matter

## References

[1] Guo L , Shorter J. Biology and pathobiology of TDP-43 and emergent therapeutic strategies. Cold Spring Harb Perspect Med. 2017;7(9):a024554. 10.1101/cshperspect.a024554 PMC5580514 27920024

[2] Jucker M , Walker LC. Self-propagation of pathogenic protein aggregates in neurodegenerative diseases. Nature. 2013;501(7465):45–51. 24005412 10.1038/nature12481 PMC3963807

[3] Lee EB , Lee VMY, Trojanowski JQ. Gains or losses: Molecular mechanisms of TDP43-mediated neurodegeneration. Nat Rev Neurosci. 2012;13(1):38–50. 10.1038/nrn3121 PMC3285250 22127299

[4] Porta S , Xu Y, Restrepo CR, et al Patient-derived frontotemporal lobar degeneration brain extracts induce formation and spreading of TDP-43 pathology in vivo. Nat Commun. 2018;9(1):4220. 30310141 10.1038/s41467-018-06548-9 PMC6181940

[5] Spillantini MG , Goedert M. Tau pathology and neurodegeneration. Lancet Neurol. 2013;12(6):609–622. 23684085 10.1016/S1474-4422(13)70090-5

[6] Brettschneider J , Tredici KD, Lee VMY, Trojanowski JQ. Spreading of pathology in neurodegenerative diseases: A focus on human studies. Nat Rev Neurosci. 2015;16(2):109–120. 25588378 10.1038/nrn3887 PMC4312418

[7] Braak H , Braak E. Neuropathological stageing of Alzheimer-related changes. Acta Neuropathol. 1991;82(4):239–259. 1759558 10.1007/BF00308809

[8] Braak H , Thal DR, Ghebremedhin E, Tredici KD. Stages of the pathologic process in Alzheimer disease: Age categories from 1 to 100 years. J Neuropathol Exp Neurol. 2011;70(11):960–969. 22002422 10.1097/NEN.0b013e318232a379

[9] James BD , Wilson RS, Boyle PA, Trojanowski JQ, Bennett DA, Schneider JA. TDP-43 stage, mixed pathologies, and clinical Alzheimer’s-type dementia. Brain. 2016;139(11):aww224-2993. 10.1093/brain/aww224 PMC5091047 27694152

[10] Robinson JL , Lee EB, Xie SX, et al Neurodegenerative disease concomitant proteinopathies are prevalent, age-related and APOE4-associated. Brain. 2018;141(7):2181–2193. 29878075 10.1093/brain/awy146 PMC6022546

[11] Mackenzie IR , Neumann M, Bigio EH, et al Nomenclature and nosology for neuropathologic subtypes of frontotemporal lobar degeneration: An update. Acta Neuropathol. 2010;119(1):1–4. 19924424 10.1007/s00401-009-0612-2 PMC2799633

[12] Brettschneider J , Tredici KD, Toledo JB, et al Stages of pTDP-43 pathology in amyotrophic lateral sclerosis. Ann Neurol. 2013;74(1):20–38. 23686809 10.1002/ana.23937 PMC3785076

[13] Brettschneider J , Tredici KD, Irwin DJ, et al Sequential distribution of pTDP-43 pathology in behavioral variant frontotemporal dementia (bvFTD). Acta Neuropathol. 2014;127(3):423–439. 24407427 10.1007/s00401-013-1238-y PMC3971993

[14] Irwin DJ , Brettschneider J, McMillan CT, et al Deep clinical and neuropathological phenotyping of Pick disease. Ann Neurol. 2016;79(2):272–287. 26583316 10.1002/ana.24559 PMC4755803

[15] Kim EJ , Sidhu M, Gaus SE, et al Selective frontoinsular von Economo neuron and fork cell loss in early behavioral variant frontotemporal dementia. Cereb Cortex. 2012;22(2):251–259. 21653702 10.1093/cercor/bhr004 PMC3256403

[16] Nana AL , Sidhu M, Gaus SE, et al Neurons selectively targeted in frontotemporal dementia reveal early stage TDP-43 pathobiology. Acta Neuropathol. 2019;137(1):27–46. 30511086 10.1007/s00401-018-1942-8 PMC6339592

[17] Hodge RD , Miller JA, Novotny M, et al Transcriptomic evidence that von Economo neurons are regionally specialized extratelencephalic-projecting excitatory neurons. Nat Commun. 2020;11(1):1172. 32127543 10.1038/s41467-020-14952-3 PMC7054400

[18] Forrest SL , Kril JJ, Halliday GM. Cellular and regional vulnerability in frontotemporal tauopathies. Acta Neuropathol. 2019;138(5):705–727. 31203391 10.1007/s00401-019-02035-7

[19] Seeley WW . Mapping neurodegenerative disease onset and progression. Cold Spring Harb Perspect Biol. 2017;9(8):a023622. 10.1101/cshperspect.a023622 PMC5538416 28289062

[20] Giannini LAA , Peterson C, Ohm D, et al Frontotemporal lobar degeneration proteinopathies have disparate microscopic patterns of white and grey matter pathology. Acta Neuropathol Commun. 2021;9(1):30. 33622418 10.1186/s40478-021-01129-2 PMC7901087

[21] Ohm DT , Cousins KAQ, Xie SX, et al Signature laminar distributions of pathology in frontotemporal lobar degeneration. Acta Neuropathol. 2022;143(3):363–382. 34997851 10.1007/s00401-021-02402-3 PMC8858288

[22] Forman MS , Zhukareva V, Bergeron C. Signature tau neuropathology in gray and white matter of corticobasal degeneration. Am J Pathol. 2002;160(6):2045–2053. 12057909 10.1016/S0002-9440(10)61154-6 PMC1850831

[23] McMillan CT , Irwin DJ, Avants BB, et al White matter imaging helps dissociate tau from TDP-43 in frontotemporal lobar degeneration. J Neurol Neurosurg Psychiatry. 2013;84(9):949–955. 23475817 10.1136/jnnp-2012-304418 PMC3737288

[24] Kim EJ , Hwang JHL, Gaus SE, et al Evidence of corticofugal tau spreading in patients with frontotemporal dementia. Acta Neuropathol. 2020;139(1):27–43. 31542807 10.1007/s00401-019-02075-z PMC7012377

[25] Whitwell JL , Josephs KA. Neuroimaging in frontotemporal lobar degeneration–predicting molecular pathology. Nat Rev Neurol. 2012;8(3):131–142. 22290573 10.1038/nrneurol.2012.7

[26] Bocchetta M , del Espinosa MI, Lashley T, Warren JD, Rohrer JD. In vivo staging of frontotemporal lobar degeneration TDP-43 type C pathology. Alzheimer’s Res Ther. 2020;12(1):34. 32220237 10.1186/s13195-020-00600-x PMC7102433

[27] Giannini LAA , Xie SX, McMillan CT, et al Divergent patterns of TDP-43 and tau pathologies in primary progressive aphasia. Ann Neurol. 2019;85(5):630–643. 30851133 10.1002/ana.25465 PMC6538935

[28] Irwin DJ , McMillan CT, Xie SX, et al Asymmetry of post-mortem neuropathology in behavioural-variant frontotemporal dementia. Brain. 2017;141(1):288–301. 10.1093/brain/awx319 PMC5837322 29228211

[29] Burke SE , Phillips JS, Olm CA, et al Phases of volume loss in patients with known frontotemporal lobar degeneration spectrum pathology. Neurobiol Aging. 2022;113:95–107. 35325815 10.1016/j.neurobiolaging.2022.02.007 PMC9241163

[30] Whitwell JL , Weigand SD, Boeve BF, et al Neuroimaging signatures of frontotemporal dementia genetics: C9ORF72, tau, progranulin and sporadics. Brain. 2012;135(Pt 3):794–806. 22366795 10.1093/brain/aws001 PMC3286334

[31] Bejanin A , Tammewar G, Marx G, et al Longitudinal structural and metabolic changes in frontotemporal dementia. Neurology. 2020;95(2):e140–e154. 32591470 10.1212/WNL.0000000000009760 PMC7455324

[32] Bullmore E , Sporns O. Complex brain networks: Graph theoretical analysis of structural and functional systems. Nat Rev Neurosci. 2009;10(3):186–198. 19190637 10.1038/nrn2575

[33] Crossley NA , Mechelli A, Scott J, et al The hubs of the human connectome are generally implicated in the anatomy of brain disorders. Brain. 2014;137(Pt 8):2382–2395. 25057133 10.1093/brain/awu132 PMC4107735

[34] Fornito A , Zalesky A, Breakspear M. The connectomics of brain disorders. Nat Rev Neurosci. 2015;16(3):159–172. 25697159 10.1038/nrn3901

[35] Toledo JB , Deerlin VMV, Lee EB, et al A platform for discovery: The University of Pennsylvania Integrated Neurodegenerative Disease Biobank. Alzheimer’s Dement. 2014;10:477–484.e1. 23978324 10.1016/j.jalz.2013.06.003 PMC3933464

[36] Mackenzie IRA , Neumann M, Baborie A, et al A harmonized classification system for FTLD-TDP pathology. Acta Neuropathol. 2011;122(1):111–113. 21644037 10.1007/s00401-011-0845-8 PMC3285143

[37] Lee EB , Porta S, Baer GM, et al Expansion of the classification of FTLD-TDP: Distinct pathology associated with rapidly progressive frontotemporal degeneration. Acta Neuropathol. 2017;134(1):65–78. 28130640 10.1007/s00401-017-1679-9 PMC5521959

[38] Capozzo R , Sassi C, Hammer MB, et al Clinical and genetic analyses of familial and sporadic frontotemporal dementia patients in Southern Italy. Alzheimer’s Dement. 2017;13(8):858–869. 28264768 10.1016/j.jalz.2017.01.011 PMC6232845

[39] Janssen JC , Warrington EK, Morris HR, et al Clinical features of frontotemporal dementia due to the intronic tau 10&plus; 16 mutation. Neurology. 2002;58(8):1161–1168. 11971081 10.1212/wnl.58.8.1161

[40] Abrahams S , Leigh PN, Goldstein LH. Cognitive change in ALS: A prospective study. Neurology. 2005;64(7):1222–1226. 15824350 10.1212/01.WNL.0000156519.41681.27

[41] Tustison NJ , Cook PA, Klein A, et al Large-scale evaluation of ANTs and FreeSurfer cortical thickness measurements. NeuroImage. 2014;99(0):166–179. 24879923 10.1016/j.neuroimage.2014.05.044

[42] Marcus DS , Wang TH, Parker J, Csernansky JG, Morris JC, Buckner RL. Open Access Series of Imaging Studies (OASIS): Cross-sectional MRI data in young, middle aged, nondemented, and demented older adults. J Cogn Neurosci. 2007;19(9):1498–1507. 17714011 10.1162/jocn.2007.19.9.1498

[43] Avants BB , Epstein CL, Grossman M, Gee JC. Symmetric diffeomorphic image registration with cross-correlation: Evaluating automated labeling of elderly and neurodegenerative brain. Med Image Anal. 2008;12(1):26–41. 17659998 10.1016/j.media.2007.06.004 PMC2276735

[44] Avants BB , Tustison NJ, Wu J, Cook PA, Gee JC. An open source multivariate framework for n-tissue segmentation with evaluation on public data. Neuroinformatics. 2011;9(4):381–400. 21373993 10.1007/s12021-011-9109-y PMC3297199

[45] Cook PA , Bai Y, Nadjati-Gilani S, et al Camino: Open-source diffusion-MRI reconstruction and processing. In: 14th Scientific Meeting of the International Society for Magnetic Resonance in Medicine. 2006:2759.

[46] Salvador R , Pena A, Menon DK, Carpenter TA, Pickard JD, Bullmore ET. Formal characterization and extension of the linearized diffusion tensor model. Hum Brain Mapp. 2005;24:144–155. 15468122 10.1002/hbm.20076 PMC6871750

[47] Daducci A , Gerhard S, Griffa A, et al The Connectome Mapper: An open-source processing pipeline to map connectomes with MRI. PLoS One. 2012;7(12):e48121. 10.1371/journal.pone.0048121 PMC3525592 23272041

[48] Maier-Hein KH , Neher PF, Houde JC, et al The challenge of mapping the human connectome based on diffusion tractography. Nat Commun. 2017;8(1):1349. 29116093 10.1038/s41467-017-01285-x PMC5677006

[49] Wang H , Suh JW, Das SR, Pluta JB, Craige C, Yushkevich PA. Multi-atlas segmentation with joint label fusion. IEEE Trans Pattern Anal Mach Intell. 2013;35(3):611–623. 22732662 10.1109/TPAMI.2012.143 PMC3864549

[50] Klein A , Tourville J. 101 labeled brain images and a consistent human cortical labeling protocol. Front Neurosci. 2012;6:171. 23227001 10.3389/fnins.2012.00171 PMC3514540

[51] Joie RL , Perrotin A, Barr L, et al Region-specific hierarchy between atrophy, hypometabolism, and Œ≤-amyloid (AŒ≤) load in Alzheimer’s disease dementia. J Neurosci. 2012;32(46):16265–16273. 23152610 10.1523/JNEUROSCI.2170-12.2012 PMC6794030

[52] Latora V , Marchiori M. Efficient behavior of small-world networks. Phys Rev Lett. 2001;87(19):198701. 10.1103/PhysRevLett.87.198701 11690461

[53] Barrat A , Barthélemy M, Pastor-Satorras R, Vespignani A. The architecture of complex weighted networks. Proc Natal Acad Sci USA. 2004;101(11):3747–3752. 10.1073/pnas.0400087101 PMC374315 15007165

[54] Freeman L . A set of measures of centrality based on betweenness. Sociometry. 1977;15:35–41.

[55] Rubinov M , Sporns O. Complex network measures of brain connectivity: Uses and interpretations. NeuroImage. 2010;52(3):1059–1069. 19819337 10.1016/j.neuroimage.2009.10.003

[56] van den Heuvel MP , Sporns O. Network hubs in the human brain. Trends Cogn Sci. 2013;17(12):683–696. 24231140 10.1016/j.tics.2013.09.012

[57] Irwin DJ , Cohen TJ, Grossman M, et al Acetylated tau neuropathology in sporadic and hereditary tauopathies. Am J Pathol. 2013;183(2):344–351. 23885714 10.1016/j.ajpath.2013.04.025 PMC3730769

[58] Irwin DJ , Byrne MD, McMillan CT, et al Semi-automated digital image analysis of Pick’s disease and TDP-43 proteinopathy. J Histochem Cytochem. 2016;64(1):54–66. 26538548 10.1369/0022155415614303 PMC4810792

[59] Spotorno N , Coughlin DG, Olm CA, et al Tau pathology associates with in vivo cortical thinning in Lewy body disorders. Ann Clin Transl Neur. 2020;7(12):2342–2355. 10.1002/acn3.51183 PMC7732256 33108692

[60] van den Heuvel MP , Sporns O. Rich-club organization of the human connectome. J Neurosci. 2011;31(44):15775–15786. 22049421 10.1523/JNEUROSCI.3539-11.2011 PMC6623027

[61] Nigro S , Tafuri B, Urso D, et al Altered structural brain networks in linguistic variants of frontotemporal dementia. Brain Imaging Behav. 2022;16(3):1113–1122. 34755293 10.1007/s11682-021-00560-2 PMC9107413

[62] Agosta F , Sala S, Valsasina P, et al Brain network connectivity assessed using graph theory in frontotemporal dementia. Neurology. 2013;81(2):134–143. 23719145 10.1212/WNL.0b013e31829a33f8

[63] Nigro S , Tafuri B, Urso D, et al Brain structural covariance networks in behavioral variant of frontotemporal dementia. Brain Sci. 2021;11(2):192. 33557411 10.3390/brainsci11020192 PMC7915789

[64] Reyes P , Ortega-Merchan MP, Rueda A, et al Functional connectivity changes in behavioral, semantic, and nonfluent variants of frontotemporal dementia. Behav Neurol. 2018;2018:9684129. 10.1155/2018/9684129 PMC5902123 29808100

[65] Agosta F , Galantucci S, Valsasina P, et al Disrupted brain connectome in semantic variant of primary progressive aphasia. Neurobiol Aging. 2014;35(11):2646–2655. 24970567 10.1016/j.neurobiolaging.2014.05.017

[66] Filippi M , Basaia S, Canu E, et al Brain network connectivity differs in early-onset neurodegenerative dementia. Neurology. 2017;89(17):1764–1772. 28954876 10.1212/WNL.0000000000004577 PMC5664301

[67] Ahmed Z , Cooper J, Murray TK, et al A novel in vivo model of tau propagation with rapid and progressive neurofibrillary tangle pathology: The pattern of spread is determined by connectivity, not proximity. Acta Neuropathol. 2014;127(5):667–683. 24531916 10.1007/s00401-014-1254-6 PMC4252866

[68] Iba M , McBride JD, Guo JL, Zhang B, Trojanowski JQ, Lee VMY. Tau pathology spread in PS19 tau transgenic mice following locus coeruleus (LC) injections of synthetic tau fibrils is determined by the LC’s afferent and efferent connections. Acta Neuropathol. 2015;130(3):349–362. 26150341 10.1007/s00401-015-1458-4 PMC4545685

[69] Clavaguera F , Hench J, Goedert M, Tolnay M. Invited review: Prion-like transmission and spreading of tau pathology. Neuropath Appl Neuro. 2015;41(1):47–58. 10.1111/nan.12197 25399729

[70] Goedert M , Eisenberg DS, Crowther RA. Propagation of tau aggregates and neurodegeneration. Annu Rev Neurosci. 2017;40(1):189–210. 28772101 10.1146/annurev-neuro-072116-031153

[71] Brown JA , Deng J, Neuhaus J, et al Patient-tailored, connectivity-based forecasts of spreading brain atrophy. Neuron. 2019;104(5):856–868.e5. 31623919 10.1016/j.neuron.2019.08.037 PMC7012373

[72] Ranasinghe KG , Hinkley LB, Beagle AJ, et al Distinct spatiotemporal patterns of neuronal functional connectivity in primary progressive aphasia variants. Brain. 2017;140(10):2737–2751. 28969381 10.1093/brain/awx217 PMC5841154

[73] Mandelli ML , Welch AE, Vilaplana E, et al Altered topology of the functional speech production network in non-fluent/agrammatic variant of PPA. Cortex. 2018;108:252–264. 30292076 10.1016/j.cortex.2018.08.002 PMC6317366

[74] Spinelli EG , Mandelli ML, Miller ZA, et al Typical and atypical pathology in primary progressive aphasia variants. Ann Neurol. 2017;81(3):430–443. 28133816 10.1002/ana.24885 PMC5421819

[75] Chen M , Ohm DT, Phillips JS, et al Divergent histopathological networks of frontotemporal degeneration proteinopathy subytpes. J Neurosci. 2022;42(18):3868–3877. 35318284 10.1523/JNEUROSCI.2061-21.2022 PMC9087810

[76] Narasimhan S , Guo JL, Changolkar L, et al Pathological tau strains from human brains recapitulate the diversity of tauopathies in nontransgenic mouse brain. J Neurosci. 2017;37(47):11406–11423. 29054878 10.1523/JNEUROSCI.1230-17.2017 PMC5700423

