# Supplementary material for: Antemortem network analysis of spreading pathology in autopsy-confirmed frontotemporal degeneration
Source: Brain Commun. 2023 May 12;5(3):fcad147. doi: 10.1093/braincomms/fcad147 (PMC10202556; doi:10.1093/braincomms/fcad147)
Supplement: fcad147_Supplementary_Data [file fcad147_supplementary_data.pdf]

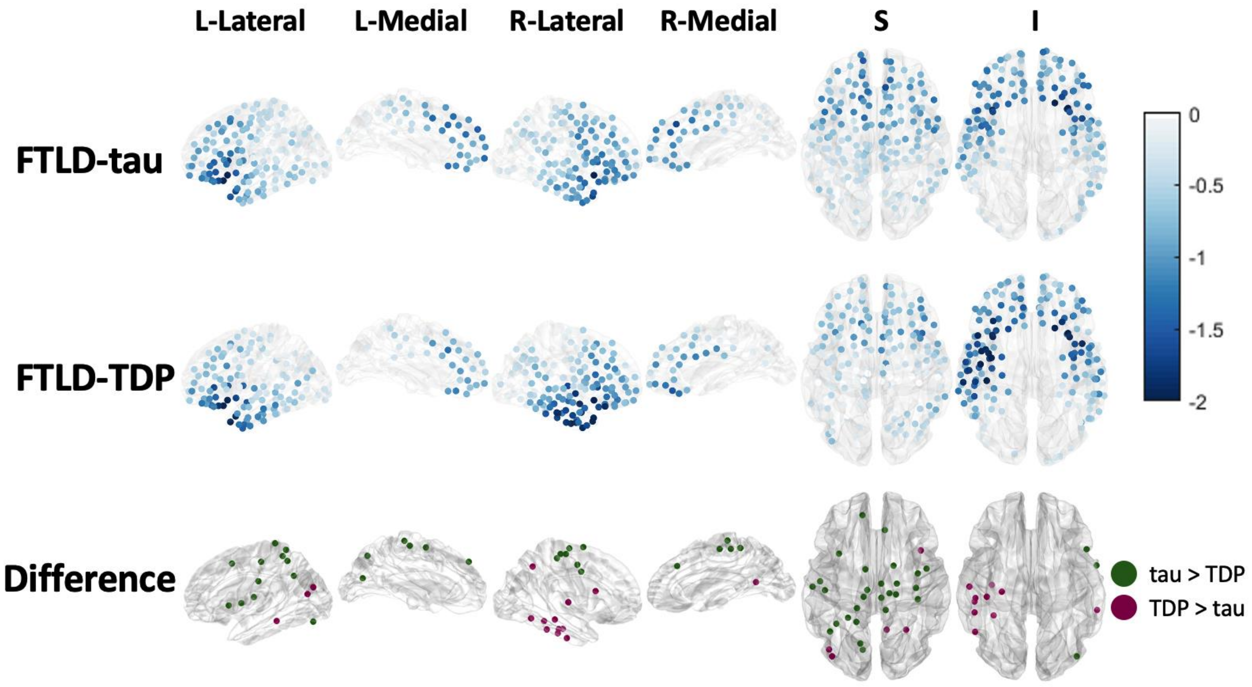

Supplementary Figure 1. Cortical atrophy distributions. Top two rows display the mean w-scores of cortical volumes at each region for the FTLD-tau and FTLD-TDP cohorts, showing the atrophy distribution for each pathology. Bottom row indicates the regions where statistically significant differences between the groups are found through one-tailed Welch's t-tests on the w-scores ($t(39)$, $p < .05$, n = 28 FTLD-tau, 13 FTLD-TDP).

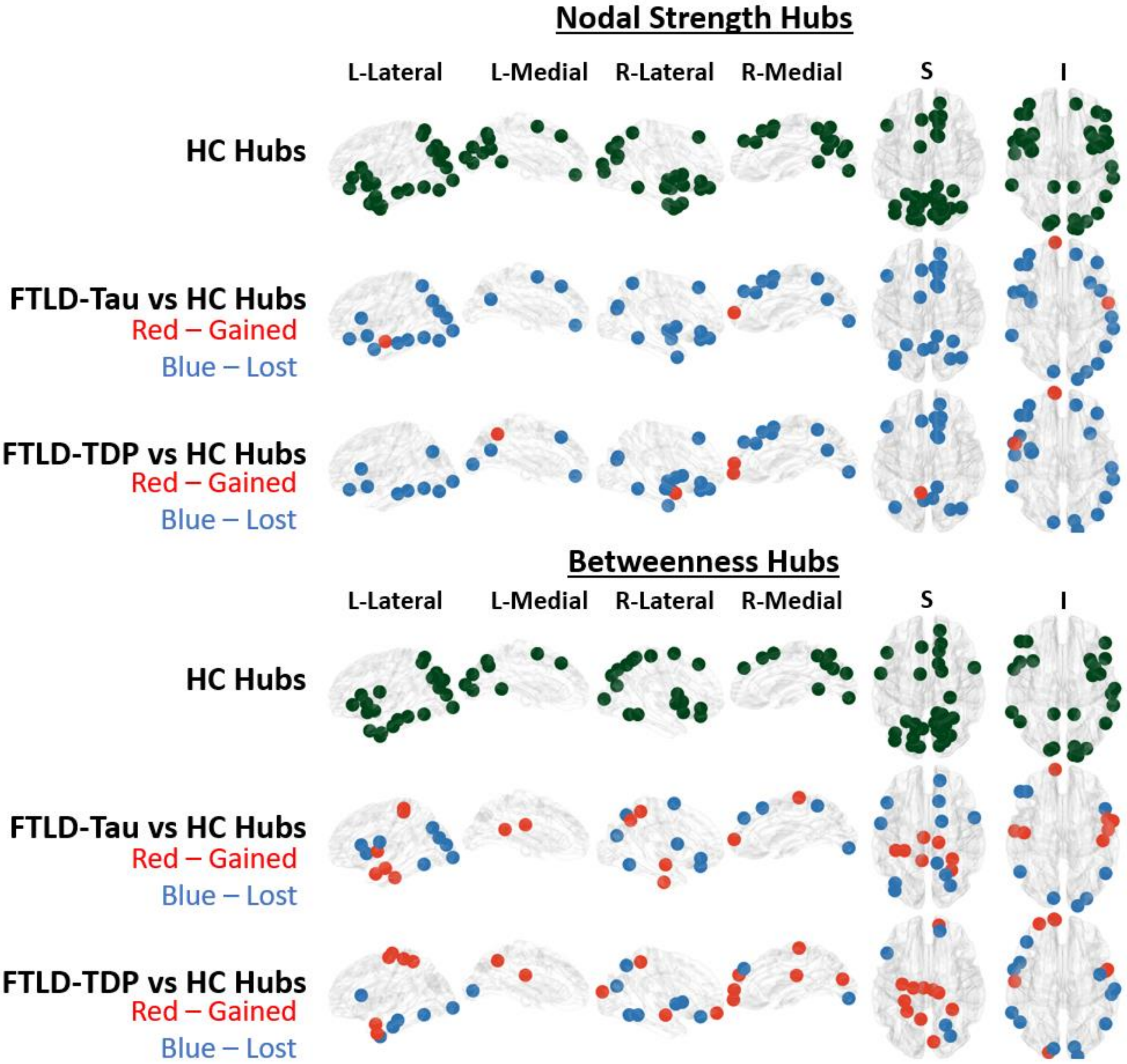

Supplementary Figure 2. Hubs differences when using nodal strength versus betweenness centrality. Shown are the relative locations of control (HC) hubs (dark green), the hubs lost (blue) and gained (red) for FTLD-tau and FTLD-TDP, when using nodal strength (top) or betweenness centrality (bottom) to determine the hubs. (n = 27 HC, 28 FTLD-tau, 13 FTLD-TDP)

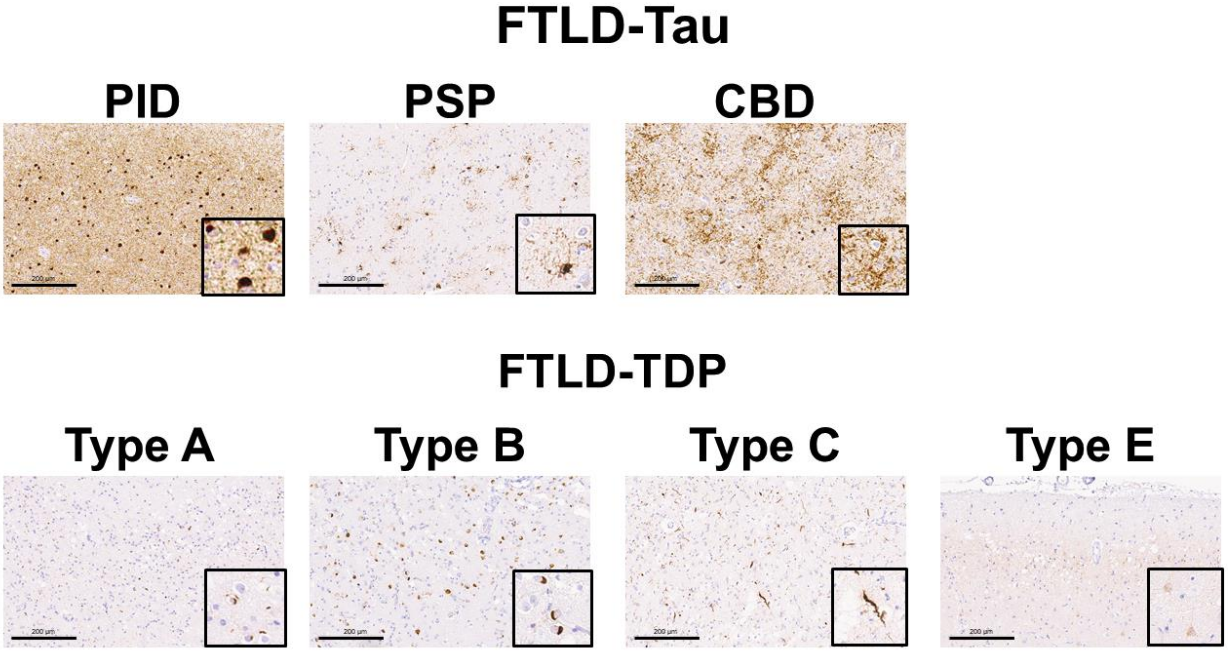

Supplementary Figure 3. Representative histopathology images of FTLD-tau and FTLD-TDP pathology from our dataset. Images were sampled from the mid-frontal cortex (BA46) in FTLD-Tau subtypes (corticobasal degeneration, CBD; Pick's disease, PiD; progressive supranuclear palsy, PSP) and FTLD-TDP subtypes (TDP A, B, C, E).
